# Epigenetic profiling reveals key super-enhancer networks driving oncogenesis in HPV-positive HNSCC

**DOI:** 10.1016/j.isci.2025.113911

**Published:** 2025-10-30

**Authors:** Fernando T. Zamuner, Spencer S. Chan, Michael D. Kessler, Ilya E. Vorontsov, Andrey Loginov, Rossin Erbe, Eddie Imada, Deborah X. Xie, Theresa Guo, Elana J. Fertig, Ivan V. Kulakovskiy, Ludmila Danilova, Alexander V. Favorov, Daria A. Gaykalova

**Affiliations:** 1 Department of Otolaryngology–Head and Neck Surgery, Johns Hopkins University School of Medicine, Baltimore, MD 21287, USA; 2 Center for Bioinformatics and Computational Biology, University of Maryland, College Park, MD 20910, USA; 3 Department of Computer Science, University of Maryland, College Park, MD 20910, USA; 4 Institute for Genome Sciences, University of Maryland School of Medicine, Baltimore, MD 21201, USA; 5 Department of Otorhinolaryngology–Head and Neck Surgery, Marlene and Stewart Greenebaum Comprehensive Cancer Center, University of Maryland Medical Center, Baltimore, MD 21201, USA; 6 Vavilov Institute of General Genetics, Russian Academy of Sciences, Moscow 119991, Russia; 7 Institute of Protein Research, Russian Academy of Sciences, Pushchino 142290, Russia; 8 Institute of Biochemistry and Genetics, Ufa Federal Research Centre of Russian Academy of Sciences, Ufa 450054, Russia; 9 Department of Oncology, Sidney Kimmel Comprehensive Cancer Center, Johns Hopkins University, Baltimore, MD 21231, USA; 10 Department of Pathology and Laboratory Medicine, Weill Cornell Medicine, New York, NY 10065, USA; 11 Department of Otolaryngology–Head and Neck Surgery, University of California, San Diego Health, La Jolla, San Diego, CA 92037, USA; 12 Moores Cancer Center, University of California, San Diego Health, La Jolla, San Diego, CA 92037, USA; 13 Sidney Kimmel Comprehensive Cancer Center, Johns Hopkins University School of Medicine, Baltimore, MD 21231, USA

**Keywords:** Epigenetics, Molecular network, Bioinformatics, Cancer

## Abstract

Human papillomavirus-positive (HPV+) head and neck squamous cell carcinoma (HNSCC) is a growing subset of cancer cases distinct from HPV-negative by fewer genetic mutations and prevalent epigenetic dysregulation. We mapped H3K27ac-marked super-enhancers (SEs) via ChIP-seq in HPV+ patient-derived xenografts (PDXs) and normal oropharyngeal mucosa, identifying tumor-specific SE domains (T-SEDs) enriched for transcription factors (TFs) including TP63, FOSL1, and JUND. These SE-associated TFs regulate key oncogenic pathways and are downregulated by BRD4 inhibition with JQ1, highlighting sensitivity to epigenetic modulation. RNA-seq data revealed coordinated dysregulation of enhancer RNAs and mRNAs near T-SEDs, linked to upregulated pathways including epithelial-mesenchymal transition and E2F targets. JQ1 treatment significantly repressed these tumor-specific pathways, suggesting a therapeutic potential for targeting SE-driven transcription in HPV+ HNSCC. This study underscores the critical role of SEs in epigenetic and transcriptional dysregulation in HPV+ HNSCC, revealing therapeutic targets and providing a framework for future mechanistic studies in this area.

## Highlights

• T-SEDs in HPV-positive HNSCC are enriched with oncogenic TFs
• Key TFs TP63, FOSL1, and JUND are regulated by HNSCC-specific SEs
• Genes near T-SEDs show significant expression changes following BRD4 inhibition
• Disruption of SE-regulated TFs reveals potential therapeutic targets in HPV+ HNSCC

## Graphical abstract

**Figure:**
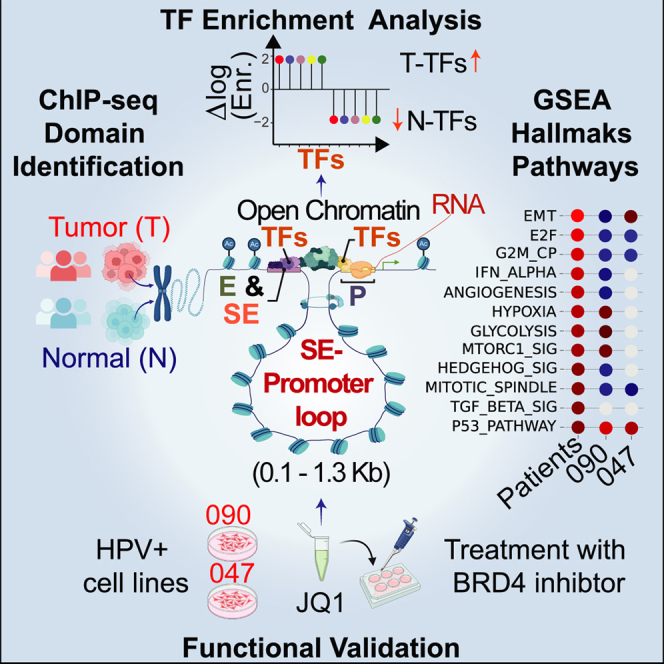

## Introduction

Head and neck squamous cell carcinoma (HNSCC) is the sixth most common cancer worldwide.^1^ Approximately one-third of HNSCC cases are caused by sexually transmitted high-risk human papillomavirus (HPV) infections.^2^ HPV-positive (HPV+) HNSCC is one of the most rapidly growing cancer populations and is represented by relatively younger and fewer smoking patients than HPV-negative (HPV–) HNSCC. Despite a better survival rate, treatment often results in functional morbidity and cosmetic deformities, significantly diminishing the quality of life for this younger population of cancer patients.^3^ While high-throughput techniques have revealed limited genetic and epigenetic alterations in HPV+ HNSCC,^4^^,^^5^^,^^6^^,^^7^^,^^8^^,^^9^ alterations in the epigenetic landscape, encompassing DNA methylation and chromatin accessibility, play a pervasive role in HNSCC oncogenesis.^10^^,^^11^^,^^12^^,^^13^ Indeed, we have recently demonstrated that chromatin remodeling contributes to HNSCC carcinogenesis.^9^^,^^14^^,^^15^^,^^16^^,^^17^^,^^18^^,^^19^

Enhancers are pivotal epigenetic elements in the regulation of gene expression, serving as platforms for the assembly of transcription regulatory complexes. These elements are densely occupied by transcription factors (TFs), co-activators, and chromatin regulators, including BRD4, that stabilize enhancer-promoter loops and facilitate transcriptional initiation complex.^20^ Distinct histone modifications characterize enhancers, particularly the acetylation of histone H3 at lysine 27 (H3K27ac) and monomethylation at lysine 4 (H3K4me1), which marks active enhancer regions.^21^^,^^22^ H3K4me3 has also been linked to active enhancers, suggesting a complex interplay of histone marks in enhancer identification and function.^23^ Among these, BRD4, a protein that recognizes the H3K27ac modifications of the enhancer region, acts as a reader by recruiting additional TFs and the mediator complex subunit MED1, thereby facilitating the assembly of the initiation complex. Additionally, enhancers often exhibit dynamic changes in histone marks, such as the cyclic addition and removal of H3K27ac, reflecting their role in fine-tuning gene expression in response to cellular signals.^24^

BRD4 is frequently overexpressed in HNSCC, promoting cancer progression through oncogene activation. Inhibition of BRD4 by JQ1 disrupts its interaction with acetylated histones, blocking the recruitment of transcriptional machinery and subsequently reducing the expression of genes critical for tumor growth. Studies have shown that inhibiting BRD4 with JQ1 disassembles the protein complexes formed by BRD4, MED1, and other TFs and epigenetic regulators. This destabilizes enhancer-promoter loops and collapses enhancer-driven transcriptional regulation, significantly affecting the expression of genes critical for tumor growth and survival.^25^^,^^26^^,^^27^

Promoters and enhancers critically influence gene expression regulation by recruiting specific TFs to transcription factor binding sites.^28^ Although the location of enhancers in genomic DNA is constant, their activity is tissue-specific and characterized by the binding of tissue-specific TFs called master TFs.^22^^,^^29^^,^^30^ Conglomerates of enhancers, enriched with binding sites for master TFs and known as super-enhancers (SEs), can transcriptionally control multiple target genes.^30^ SEs can reach out to hundreds of kilobases and regulate transcriptional activation of their in-cis target genes over several megabases away through the formation of chromatin loops.^10^^,^^28^^,^^31^^,^^32^^,^^33^^,^^34^^,^^35^ SEs are characterized by a higher occupancy of transcription mediators, NIPBL, P300, CHD7, BRD4, KLF4, and cohesin compared to typical enhancers.^22^^,^^30^ Also, SEs were recently identified as critical epigenetic regulators of gene expression during cell differentiation and cancer development.^10^^,^^21^^,^^22^^,^^28^^,^^30^^,^^36^^,^^37^^,^^38^ Indeed, studies have shown that most oncogenes are governed by actively transcribing enhancers and SEs.^22^^,^^29^^,^^39^

TFs are pivotal regulators of gene expression, orchestrating processes by binding to specific DNA sequences within the promoter, enhancer, and SE regions.^40^ Previously, we used the TRANSFAC database^41^ to examine TF activity in head and neck cancer, exploring regulatory mechanisms between HPV+ and HPV−.^42^ To identify TFs involved in HPV+ HNSCC and specifically active in normal or tumor tissues, in this study, we performed TF binding sites enrichment analysis with an in-house pipeline. Compared to a pure motif scanning approach, such as HOMER,^43^ we decided to rely on actual ChIP-seq data, which provides an extra layer of evidence of TF binding and, consequently, specificity of predictions. There are multiple alternative collections, such as ReMAP,^44^ ChIPAtlas,^45^ or GTRD,^46^^,^^47^ containing reprocessed ChIP-seq data for human TFs. Here, we employed a detailed map of TF-DNA interactions, the cistrome,^48^ built on top of a wide compendium of published ChIP-seq data reprocessed in GTRD^47^ (GTRD, http://gtrd.biouml.org).

The spatial organization of SEs relative to their target genes is crucial for their regulatory function. SEs can influence gene expression over long genomic distances by forming chromatin loops that bring enhancers close to promoters, facilitating the efficient recruitment of transcriptional machinery.^49^^,^^50^ Disruption of these loops can impair gene regulation, highlighting the importance of SE spatial organization in maintaining normal cellular functions.^49^

Recent studies have revealed that enhancers can trigger the transcription of non-coding RNAs, termed enhancer RNAs (eRNAs),^51^^,^^52^^,^^53^^,^^54^^,^^55^ through RNA polymerase II activity.^56^ These eRNAs establish connections with promoters, facilitating the formation of enhancer-promoter (E-P) loops that initiate gene expression,^57^ serving as markers of enhancer activity.^58^ Notably, the FANTOM consortium has identified 43,011 active enhancers capable of transcribing eRNAs.^59^^,^^60^ Furthermore, dysregulation of gene expression in cancer is often linked to the activation of specific signaling pathways that drive tumor growth and survival, and SE plays a crucial role in this process.^22^^,^^39^^,^^61^

Despite significant advancements, the role of SEs and their involvement with TFs, bromodomain inhibitors, eRNAs, and dysregulated pathways in HPV+ HNSCC remains poorly understood. Addressing this gap requires an integrative analysis of epigenetic and transcriptional data in biologically relevant models. However, chromatin immunoprecipitation (ChIP) of primary HPV+ tumors poses technical challenges due to the limited amounts of viable tissue typically obtained during surgical resection or biopsy. To accommodate these constraints, the ENCODE v4 histone ChIP-seq guidelines prioritize quality control metrics, such as replicate concordance, irreproducible discovery rate (IDR), and fraction of reads in peaks (FRiP), over large sample numbers when biospecimens are scarce.^62^ Within this framework, we assembled a high-quality H3K27ac ChIP-seq dataset comprising two independent HPV+ HNSCC patient-derived xenografts (PDXs), two HPV+ cell lines, and two normal mucosal specimens (UPPP), enabling a robust, multi-model investigation of enhancer regulation in this tumor type.

In this study, we comprehensively mapped the active enhancer landscape of HPV+ HNSCC with a particular focus on SEs. We identified tumor-specific super-enhancer domains (T-SEDs) enriched for key TFs, including TP63, FOSL1, and JUND, and demonstrated that these domains regulate genes critical to oncogenic signaling and proliferation. Notably, BET bromodomain inhibition with JQ1 disrupted the expression of both viral (*E6*/*E7*) and host oncogenes proximal to T-SEDs, confirming their functional dependence on SE activity. Integration of eRNA expression, TF enrichment, and pathway analysis further revealed coordinated epigenetic and transcriptional regulation of mitotic and cell cycle programs. Together, these results define a core SE-driven transcriptional network in HPV+ HNSCC and highlight new epigenetic vulnerabilities for therapeutic targeting in this disease.

## Results

### Exploring the epigenetic landscape and gene expression dynamics in head and neck squamous cell carcinoma

Investigating the interplay between epigenetic regulation and gene expression in head and neck squamous cell carcinoma (HNSCC), we conducted a multi-omics analysis using high throughput epigenetics and gene expression data on tumor and normal samples, as well as on two HPV+ cell lines (UM-SCC-047 and UPCI-SCC-090) treated with JQ1 or DMSO as a control. We aimed to uncover the active chromatin landscape and its impact on transcriptional changes in this disease.

Current analysis pipelines are limited in their ability to comprehensively integrate multi-omics data, particularly when correlating epigenetic modifications with gene expression patterns in a tissue- and disease-specific context. Most existing tools lack the specificity needed to distinguish between tumor-specific and normal-specific regulatory elements, especially in complex diseases like HPV+ HNSCC, where epigenetic regulation is essential in tumorigenesis. This limitation motivated us to develop a new pipeline that enables a detailed integrative analysis of high-throughput epigenetic and gene expression data, allowing for the precise identification of active chromatin domains and their associated transcriptional changes in HNSCC.

**Figure 1:**
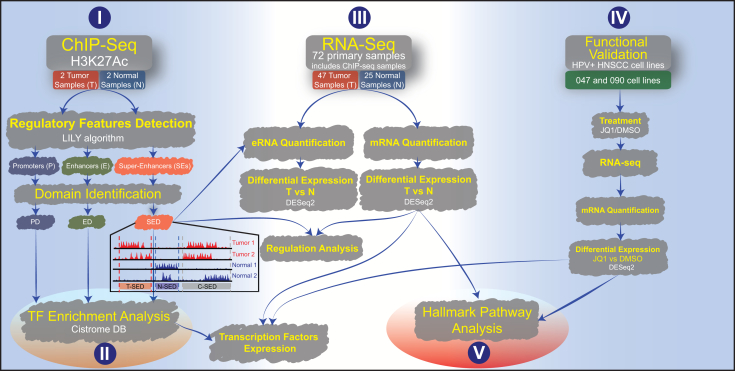
Study workflow overview (I) H3K27ac-ChIP-seq was performed on two patient-derived xenograft (PDX) tumors and two non-cancerous tissues, analyzed via the LILY algorithm to identify super-enhancers (SE), enhancers (E), and promoters (P). These elements were categorized into domains specific to the tumor (T-SED), normal (N-SED), or common to both (C-SED). (II) TF enrichment in SED, ED, and PD was analyzed using the TF cistrome, complemented by TF expression analysis from primary and cell line mRNA. (III) RNA-seq of 47 tumors and 25 normal samples quantified mRNA and eRNA expressions, with differential analysis conducted using DESeq2. (IV) Functional analysis on two HPV+ HNSCC cell lines, UM-SCC-047 and UPCI-SCC-090. RNA-Seq post-treatment with DMSO or JQ1, followed by DESeq2 for differential expression. (V) Pathway analysis using Hallmark gene sets from MSigDB was based on differentially expressed genes from both primary samples and cell lines. ChIP-seq, chromatin immunoprecipitation sequencing; H3K27ac, histone H3 lysine 27 acetylation; T, tumor; N, normal; PD, promoter domains; ED, enhancer domains; SED, super-enhancer domains; T-SED, tumor-specific super-enhancer domains; N-SED, normal-specific super-enhancer domains; C-SED, common super-enhancer domains; eRNA, enhancer RNA; DESeq2, R package to analyze differential expression.

To address these challenges, we designed a comprehensive workflow that integrates multiple layers of genomic data to map active chromatin regions and understand their regulatory roles in gene expression. Our pipeline, outlined in Figure 1, consists of (I) epigenetic analysis: In the first step (top left), we conducted H3K27ac-ChIP-seq on two patient-derived xenograft (PDX) tumor samples and two primary non-cancer tissues (normal tissues from the upper aerodigestive tract, UPPP). The ChIP-seq data were processed using the LILY algorithm to identify promoters, enhancers, and super-enhancers (P/E/SE).^63^ We detected 162,609 peaks spanning the four samples: 57,323 promoters, 101,684 enhancers, and 3,602 super-enhancers across these samples (Table S1). We combined areas that overlap into specific groups called promoter domains (PD, *n* = 17,186), enhancer domains (ED, *n* = 70,011), and super-enhancer domains (SED, *n* = 2,043) (Table S2). Domains that appeared exclusively in tumor samples were labeled as tumor-specific (T), those exclusively in normal samples were labeled as normal (N), and domains present in both tumor and normal samples were labeled as common (C). (II) Transcription factor enrichment analysis: In the bottom left, a transcription factor enrichment analysis was conducted using the cistrome dataset^48^ and aimed to define the abundance of TFBSs associated with the identified epigenetic domains (PD, ED, and SED) (*See* STAR Methods *for details*); (III) gene expression analysis: next (middle part), we performed RNA-seq analysis on a larger cohort comprising 47 HNSCC tumor samples and 25 normal samples that included two tumor and two normal samples that were used for ChIP-seq analysis at step I. We quantified the expression levels of all mRNAs and employed SED from step I to calculate the expression of enhancer RNAs (eRNAs). Differential expression analysis between tumor and normal samples was performed using DESeq2^64^ for both eRNAs and mRNAs. The genes that exhibited differential expression (mRNA) were subjected to pathway analysis using Hallmark gene sets from the molecular signatures database (MSigDB).^65^ Results of the differential mRNA expression (T vs. N) were used in the regulation, TF enrichment, and pathway analyses. (IV) Functional validation: the rightmost panel represents the functional analysis using two HPV+ HNSCC cell lines: UM-SCC-047 (047) and UPCI-SCC-090 (090). These cell lines were treated with DMSO (vehicle control) or JQ1 (an inhibitor of the BRD4 protein, which reads H3K27ac marks). RNA-seq was used to quantify mRNA expression, and differential expression analysis was conducted using DESeq2^64^ for JQ1-treated versus DMSO-treated cells; (V) hallmarks pathway analysis: differentially expressed genes identified from RNA-seq analyses across primary samples and the HPV+ cell lines were used for gene set enrichment analysis using hallmark pathways from the human MSigDB.

Our novel pipeline overcomes the limitations of existing tools by enabling a multi-dimensional integration of epigenetic marks, TF binding profiles, and gene expression data, tailored specifically for the complex regulatory environment of HNSCC. This integrative approach provides a more comprehensive understanding of how SEs shape transcriptional changes in both HNSCC and normal tissues, utilizing primary samples and cell lines.

### Transcription factor enrichment analysis of epigenetic domains reveals TF specificity in ED and SED

Distinguishing between tumor-specific and normal-specific regulatory elements is essential for advancing our understanding of the molecular mechanisms underlying tumorigenesis and normal cellular functions. Our analysis pipeline enables comprehensive analysis of TF enrichment in HPV+ HNSCC across various epigenetic domains, specifically promoter domains (PD), enhancer domains (ED), and super-enhancer domains (SED).

Global TF binding profiling of HPV+ HNSCC, e.g., with ChIP-seq, is unavailable, but compiling existing ChIP-seq data with the motif predictions provides an incomplete but reliable reference of TF binding landscape in the human genome. Here, we used the motif-annotated cistrome,^48^ which provides genomic maps of reproducible binding regions for 599 human TFs. The cistrome is based on previously published ChIP-seq data from GTRD,^46^^,^^47^ and also allows considering only the regions supported by significant binding motif occurrences of the TFs of interest (see STAR Methods). This resource allows us to precisely characterize TF binding sites near human transcription start sites (TSS), leading to accurate pinpointing of gene targets regulated by TFs. Additionally, the comprehensive nature of the Cistrome database facilitates the identification of co-regulated genes within differential gene expression datasets.

**Figure 2:**
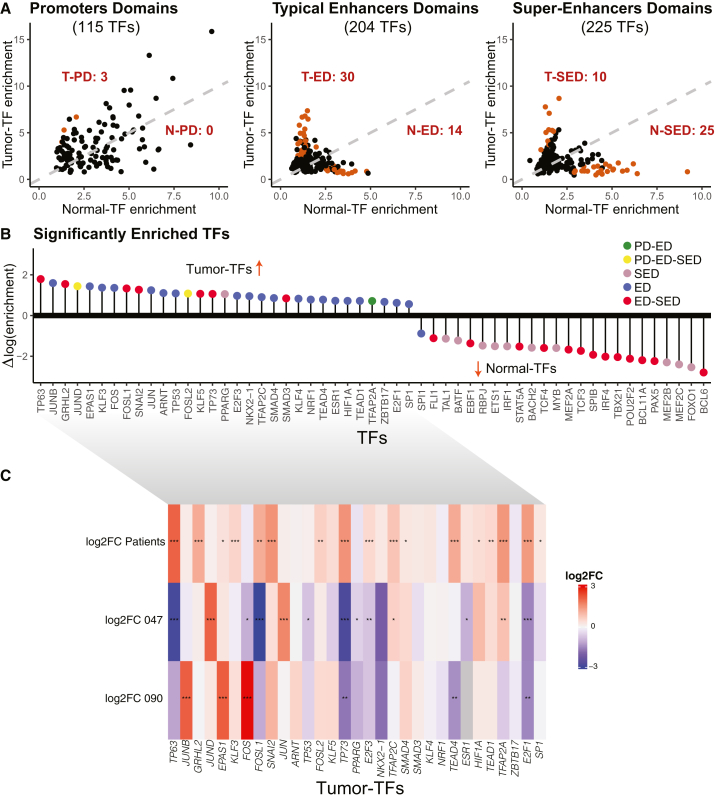
Transcription factor analysis in HNSCC (A) The transcription factor enrichment analysis was completed using the TF cistrome^58^ for PD (left), ED (middle), and SED (right), and, for every TF, the TFBS enrichment was calculated for the tumor (*y* axes) and normal (*x* axes) specific domains separately. TFs with statistically significant TFBS enrichment (Bonferroni adjusted *p* value≤ 0.05) are shown as orange dots in each graph. (B) TFs with statistically significant enrichment in tumors [Δlog(enrichment) > 0] or normals [Δlog(enrichment) < 0] are shown. Their specificity to PD, ED, and/or SED is color-coded to indicate domain types: green (promoter and enhancer), yellow (promoter, enhancer, and super-enhancer), pink (super-enhancer), blue (enhancer), and red (enhancer and super-enhancer). (C) Heatmap of the differential expression of tumor-specific TFs from B. The differential expression is shown as log2-transformed fold change (log2FC) of expression levels of TFs in 47 HPV+ HNSCC tumor vs. 25 normal samples and in two HPV+ cell lines (UM-SCC-047 [047] and UPCI-SCC-090 [090]) treated with JQ1 (treatment) vs. treated with DMSO (control). The heatmap is color-coded according to the log2FC scale. The heatmap is annotated with asterisks (∗, ∗∗, ∗∗∗) to indicate statistical significance (FDR-adjusted *p* value <0.05, <0.01, and <0.001, respectively). This figure shows TF-binding enrichment statistics only; no chromatin-loop or Hi-C data are depicted.

Our objective was to identify tumor and normal patterns of TF binding that are crucial for understanding transcriptional regulation and its impact on gene expression associated with the H3K27ac mark. In our analysis, we merged neighboring regions bound by the same transcription factor (TF) using 10k and 100k distance thresholds, resulting in data for 372 TFs. This merging process allowed us to create a more comprehensive and accurate dataset by combining information from different distance thresholds, which provided a robust basis for our analysis. We observed that the DNA regions assigned to PD, ED, and SED were enriched with TFBSs for 115, 204, and 225 TFs, respectively. Using these TFs, we calculated the TFBS enrichment for tumor-specific and normal-specific promoter domains (PD), enhancer domains (ED), and super-enhancer domains (SED), as detailed in our STAR Methods section and Table S3. TFBS. TF enrichment for each domain type is plotted in Figure 2A.

For PD, we identified 115 TFs, TFBSs for three of which were significantly enriched in tumor-specific PDs: FOSL2, JUND, and TFAP2A, whereas no significant enrichment was found in normal-specific PDs. This is visually represented in Figure 2A (left), where each point on the graph represents a TF. The distribution of points along the diagonal line suggests that the TFBS enrichment for these TFs is similar in both tumor and normal-specific domains, indicating poor tissue specificity in the binding preferences of the analyzed TFs. Detailed results and further statistical analyses are provided in Table S4.

In contrast, the ED analysis revealed 204 TFs, with 30 showing substantial tumor-specific TFBS enrichment, including TP63, FOSL1, JUND, JUNB, and KLF5, underscoring their potential roles in tumorigenesis. Conversely, 14 TFs were identified as normal-specific in ED (N-ED), such as FLI1, SPI1, and PAX5, highlighting their importance in maintaining normal cellular functions (Figure 2A, middle). Notably, there was a significant increase in the tissue specificity of the TF enrichment, and data points deviated from the symmetry diagonal observed in the ED. This is especially obvious compared to PD-specific patterns (Figure 2A, left).

In the case of SED, 225 TFs were analyzed, with TFBSs of 10 TFs identified as enriched in tumor-specific domains (T-SED), including TP63, SMAD3, JUND, and FOSL1/2. Additionally, 25 TFs showed normal-specific enrichment (N-SED), such as FLI1, TAL1, and BACH2 (Figure 2A, right). The increased tissue specificity observed in SED compared to PD and ED underscores the unique transcriptional profiles and regulatory mechanisms in these distinct domains.

### Differential enrichment of TFs on domain-specific regions reveals key regulators in HNSCC

Following the identification of significantly enriched TFs within promoter domains (PD), enhancer domains (ED), and super-enhancer domains (SED) in both tumor-specific and normal-specific regions, we investigated their differential binding patterns between tumor and normal tissues. This analysis aimed to find key TFs differentially enriched in tumor-specific domains and may drive the aberrant gene expression profiles in HPV+ HNSCC. Consequently, we identified 31 TFs predominantly enriched in tumor- and 24 TFs enriched in normal-specific domains (Figure 2B).

Notably, JUND and FOSL2 were consistently found to be significant players in all types of tumor-specific domains. Several tumor-specific TFs were detected within the ED and SED, including TP63, FOSL1, JUND, GRHL2, SNAI2, KLF5, TP73, and SMAD3. TFAP2A, on the other hand, was enriched in both PD and ED, indicating its involvement in regulating both promoter and enhancer regions. Overall, we found that the TFs binding EDs (Figure 2B) were predominant among tumor-specific TFs. Normal-specific TFs were enriched in both ED and SED, indicating that the latter may play a more substantial role in maintaining normal cellular function.

### Tumor-specific TFs exhibit enhanced expression in tumors and subsequent downregulation following JQ1 treatment

After identifying TFs with differential TFBS enrichment between tumor- and normal-specific domains, we wanted to investigate their relevant gene expression patterns. Our focus narrowed to TFs displaying tumor-specific binding within chromatin domains (PD, ED, and SED), constituting a subgroup of 31 TFs described previously (Figure 2B). Differential gene expression analysis of these 31 TFs between primary HPV+ HNSCC tumors (*n* = 47) and non-cancer control (*n* = 25) samples showed a prevalent trend of overexpression in tumor tissues. Specifically, TP63, GRHL2, EPAS1, KLF3, FOSL1, SNAIL2, FOSL2, TP73, E2F3, TFAP2C, SMAD4, TEAD4, HIF1A, TEAD1, TFAP2A, and E2F1 exhibited significantly elevated expression levels in tumors compared to normal samples (Figure 2C).

Given that our analysis relied on H3K27ac-marked epigenetic domains, we employed JQ1, a BET protein inhibitor known to bind H3K27ac. Next, we sought to elucidate the expression of these tumor-specific TFs after JQ1 treatment. UM-SCC-047 and UPCI-SCC-090 cells treated with JQ1 demonstrated a relative downregulation in the expression of *TP63*, *FOSL1*, *TP73*, *TEAD4*, *E2F1*, *TP53*, *PPARG*, and *E2F3*—TFs primarily enriched in tumor-specific domains (Figure 2C).

In summary, we have identified a set of TFs with differential enrichment and overexpression in tumor tissues compared to normal samples. Furthermore, our findings suggest a dependence of these tumor-specific TFs on the epigenetic landscape, demonstrated through their downregulation following BRD4 inhibition.

### Genes adjacent to tumor super-enhancer domains display a reduction in gene expression levels after JQ1 treatment

**Figure 3:**
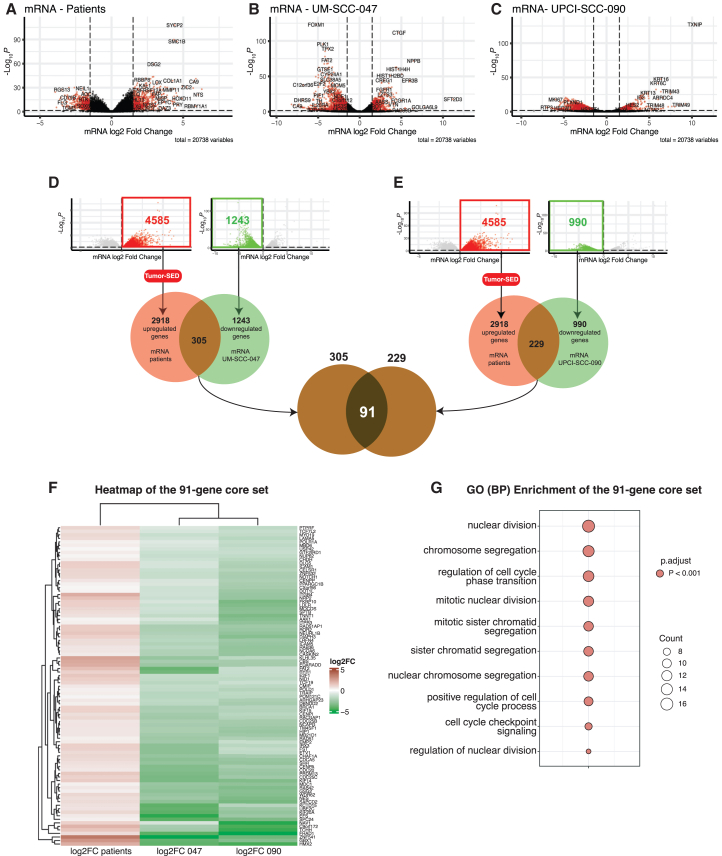
Cross-model integration identifies a core gene set regulated by tumor-specific super-enhancers and repressed by JQ1 in HPV+ HNSCC. Volcano plots in (A–C) show differential gene expression (log_2_ fold change vs. –log_10_ adjusted *p* value) for (A) HPV+ tumors vs. normal mucosa (*n* = 47 vs. 25), (B) UM-SCC-047 cells treated with JQ1 vs. DMSO, and (C) UPCI-SCC-090 cells treated under the same conditions. Red and green dots denote significantly upregulated and downregulated genes (p.adj <0.05), respectively; gray denotes non-significant genes Panels. (D and E) display Venn diagrams showing the intersection between genes that are upregulated in tumors, located near tumor-specific SEs (T-SEDs), and downregulated after JQ1 treatment in (D) UM-SCC-047 and (E) UPCI-SCC-090. This integrative analysis yields a 91-gene core set that is epigenetically activated in tumors and repressed by BET inhibition. (F) shows a heatmap of expression patterns for the 91-gene core set across tumors and both cell lines, revealing consistent upregulation in HPV+ tumors and suppression following JQ1 treatment. (G) Presents gene ontology enrichment analysis (biological process terms) for the core set, with top terms including mitotic nuclear division, chromosome segregation, and G2/M checkpoint regulation. Dot size reflects the number of genes per term; all terms shown have adjusted *p*-values <0.001.

To test the hypothesis that genes adjacent to T-SED tend to reduce their expression after the JQ1 treatment, we performed the differential expression analysis of RNA-seq data from patient samples (tumor vs. normal) and two HPV+ HNSCC cell lines (UM-SCC-047 and UPCI-SCC-090) treated with either JQ1 or DMSO (Figures 3A–3C). The differential expression analysis of patient samples revealed 4,585 genes significantly upregulated in tumor patient samples, with 2,918 genes adjacent to T-SEDs and, as we hypothesized, under the regulation of those T-SEDs.

Importantly, when we compared these genes with those downregulated after JQ1 treatment in UM-SCC-047 (*n* = 1,243), we discovered a significant negative correlation between tumor-specific gene expression (Log2FC[T/N]) and BRD4 inhibition (Log2FC[JQ1/DMSO]) (R = −0.28, p = 1e-06) (Figure S1A). This negative correlation suggested that the upregulation of genes under T-SED regulation in patient samples was directly linked to the downregulation of these genes in UM-SCC-047 after JQ1 treatment. Similarly, we compared the upregulated genes in patient samples with those downregulated after JQ1 treatment in UPCI-SCC-090 (Figure S1B). Although there was a negative correlation between the two datasets (R = −0.12), it was not statistically significant (*p* = 0.074), indicating that the trend was weaker and not conclusive in UPCI-SCC-090 compared to UM-SCC-047.

To validate the RNA-seq findings and assess the direct impact of BRD4 inhibition on viral oncogene expression, we performed RT-qPCR analysis of HPV16 *E6* and *E7* in both UM-SCC-047 and UPCI-SCC-090 cell lines following JQ1 treatment. Treatment with 500 nM JQ1 significantly decreased HPV16 *E6* and *E7* transcript levels by 50%–70% in both UM-SCC-047 and UPCI-SCC-090 cells (Figure S2; *p* < 0.01 for all comparisons vs. fold change = 1.0, one-sample t-test and Wilcoxon signed-rank test). In parallel, the host oncogene *EGFR* was concurrently downregulated by 40%–60% in both cell lines, corroborating the RNA-seq trends. *TFAP2A* expression was also significantly reduced by RT-qPCR for both cell lines; however, RNA-seq data showed significant downregulation in UPCI-SCC-090 but insignificant downregulation in UM-SCC-047.

In summary, our RNA-seq data analysis from patients and cell lines confirms that the upregulation of genes under T-SED regulation in HPV+ HNSCC patient samples is linked to their downregulation in cell lines after JQ1 treatment, especially in UM-SCC-047. These findings not only confirm our previous observations but also provide compelling additional evidence for the crucial role of JQ1 in modulating gene expression in these enhancer-regulated genes.

### Integrated analysis identifies a core set of SE-associated, JQ1-sensitive genes in HPV+ HNSCC

To define a core regulatory program associated with T-SEDs, we performed an integrative analysis by intersecting (1) genes significantly upregulated in patients (p.adj <0.05), (2) genes located near tumor-specific super-enhancer domains (T-SEDs), and (3) genes downregulated by JQ1 in both HPV+ cell lines (p.adj <0.05). This intersection yielded a set of 91 genes that are epigenetically activated in tumors, proximal to super-enhancer domains, and sensitive to BRD4 inhibition, defining a convergent SE-linked and JQ1-repressible oncogenic network (Table S5; Figures 3D and 3E).

To evaluate the statistical robustness of these associations, we performed Fisher’s exact tests comparing the overlap between tumor-upregulated, T-SED-proximal genes and those downregulated after JQ1 treatment. We observed significant enrichment for both cell lines: UM-SCC-047 (odds ratio = 2.10, 95% CI [1.83–2.41], *p* = 3.7 × 10^−24^) and UPCI-SCC-090 (odds ratio = 1.91, 95% CI [1.63–2.23], *p* = 4.3 × 10^−15^). The 91-gene core set itself showed strong enrichment (odds ratio = 1.95, 95% CI [1.52–2.49], *p* = 2.1 × 10^−7^), confirming a statistically significant convergence of super-enhancer proximity, tumor-specific activation, and BET sensitivity (Table S6).

To visualize this network, we generated a heatmap (Figure 3F), which shows consistent overexpression across HPV+ patient tumors and coordinated repression in both cell lines following JQ1 treatment. Notable members include *MYC* and *RAD51*, which play essential roles in transcriptional amplification and DNA repair, respectively, and are known HPV-associated oncogenic drivers. Other key genes, such as *CDC20*, *CCNB1*, *CHK1*, and *UBE2C*, are well-known regulators of mitotic progression and therapeutic resistance in squamous carcinomas.

Gene ontology (GO) enrichment analysis of the 91-gene set (Table S7; Figure 3G) revealed significant enrichment for mitotic and cell cycle-related processes, including *nuclear division* (16 genes, p.adj = 3.3 × 10^−7^), *chromosome segregation* (14 genes, p.adj = 3.6 × 10^−6^), *mitotic nuclear division* (12 genes, p.adj = 3.5 × 10^−6^), and *regulation of cell cycle phase transition* (13 genes, p.adj = 6.7 × 10^−5^). These annotations indicate that HPV+ tumor super-enhancers coordinate a G2/M checkpoint and chromosomal segregation program that is selectively repressed by BRD4 inhibition. Many of these genes also overlap canonical Hallmark gene sets such as G2M Checkpoint and E2F Targets, underscoring their role in sustaining proliferative signaling. These pathway-level enrichments are explored in more detail in a subsequent section.

### Defining the disease-specific activity of super-enhancer domains in HNSCC

**Figure 4:**
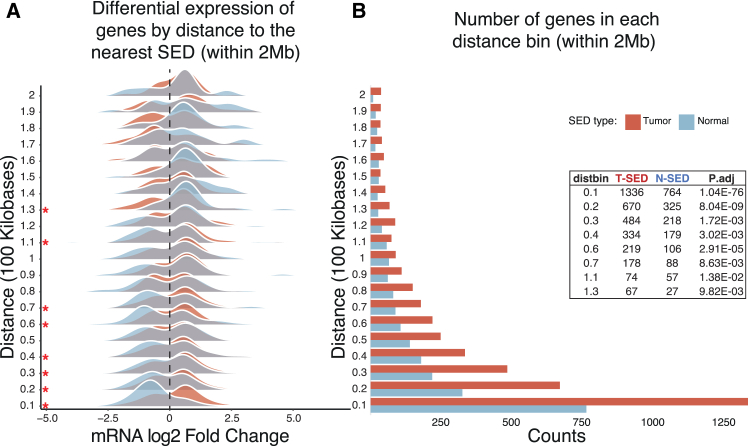
Distance analysis of super-enhancer domain (SED) activity (A) Differential expression of genes by distance to the nearest SED within a 2-megabase (Mb) range. The mRNA log2 fold change in expression in tumor vs. normal samples is plotted for genes adjacent to T-SED (red) and N-SED (blue) across 100 kilobases (Kb) increments. Density ridgeline plots illustrate the distribution of log2 fold changes, with asterisks indicating statistically significant differences from two-sided Wilcoxon rank-sum tests, with *p* values Benjamini-Hochberg (BH) adjusted across bins (reported as “p.adj” = FDR) (∗ FDR <0.05, ∗∗ FDR <0.01, ∗∗∗ FDR <0.001) between genes adjacent to T-SED and N-SED within specific distance bins. (B) Number of genes categorized according to their distance from the nearest SED. The number of genes within each 100 Kb distance bin from the nearest SED is shown for T-SED (red) and N-SED (blue). The table inset details the gene counts and BH-adjusted Wilcoxon *p* values (“p.adj”, i.e., FDR) for significant distance bins, revealing a higher concentration of genes near T-SEDs and significant differences in log2 fold change distributions between T-SED and N-SED. Significant enrichment of T-SED and N-SED is observed across different distance bins, with the strongest enrichment at approximately 0.1 Mb (p.adj: 1.04E-76) and decreasing but remaining significant at distances up to 1.3 Mb (p.adj: 9.82E-03).

To elucidate the disease-specific activity of SEDs, we analyzed differential gene expression in relation to the distance between a SED and the transcriptional start site (TSS) for each gene (Figure 4). Genes adjacent to tumor-specific or normal-specific SEDs were categorized based on their proximity to these domains and divided into sequential 100 Kb regions. We looked at the genes upstream and downstream of SED up to 2 Mb. Within each 100 Kb region, log fold change from the differential gene expression analysis of the patient samples was compared between genes adjacent to T-SED and N-SED using the two-sided Wilcoxon test (Table S8). This analysis showed that the activity of SED extended to at least 1.3 Mb, after which we utilized a ±2 Mb window to identify genes impacted by SED activity for further examination (Figure 4A). The differential expression of genes (log2 fold change) adjacent to T-SED and N-SED shows the most significant differences within the 0.1 to 0.7 Mb range from the nearest gene (except the 0.5 Mb region). Statistically significant differences, as marked by asterisks, are prevalent in bins closer to SEDs, highlighting the potential regulatory influence of these super-enhancer regions in a tumorigenic context. Beyond the 0.8 Mb distance, the differences in gene expression between genes adjacent to T-SEDs and N-SEDs diminish, suggesting that the regulatory influence of SEDs decreases with distance. This indicates that SEDs most strongly affect genes within approximately 0.8 Mb, aligning with the known long-range regulatory potential of super-enhancers.

We observed a significant concentration of genes within the closest bin (0.1 Mb) to the SEDs. Our statistical analysis revealed a marked increase in the number of genes near both T-SED and N-SED across different distance bins, with the most pronounced effect observed at approximately 0.1 Mb (*p* value: 1.04E-76) (Figure 4B). This finding indicates that genes closest to SEDs are most strongly influenced by their activity.

At distances of 0.2 Mb and beyond, we continued to observe a notable presence of genes near next to T-SED and N-SED, although the strength of the effect decreased as the distance from the SED increased (*p* values: 8.04E-09 [0.2 Mb bid] to 9.82E-03 [1.3 Mb bid]). This observation suggests that the influence of SEDs on gene expression extends beyond its immediate vicinity, affecting genes up to 1.3 Mb away.

### Differential expression analysis of enhancer RNAs (eRNAs)

**Figure 5:**
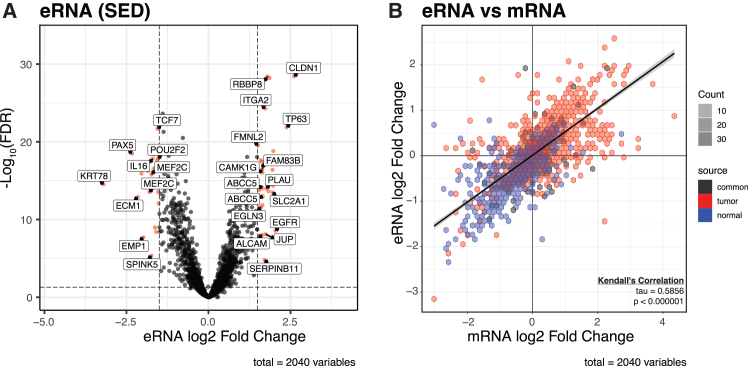
Differential expression and correlation of enhancer RNAs (eRNAs) and mRNAs in tumor and normal samples (A) Volcano plot of differential eRNA expression. The *x* axis represents the log2 fold change of eRNA expression, and the *y* axis represents the -log10 of FDR-adjusted *p* values (“p.adj”, Benjamini-Hochberg). eRNAs with significant changes are highlighted, with vertical dashed lines indicating log2 fold change thresholds (±1.5) and the horizontal dashed line representing an FDR (“p.adj”) threshold of 0.05 (plotted as -log10 for visualization). (B) Hexbin plot comparing log2 fold changes of eRNAs and mRNAs. The *x* axis represents the log2 fold change of mRNA expression, and the *y* axis represents the log2 fold change of eRNA expression. Points are color-coded by sample source: red for tumor, blue for normal, and black for common SEDs. The color saturation inside the hexagons indicates the count, reflecting the frequency or abundance of the data points. The correlation between eRNA and mRNA expression changes is shown, with Kendall’s Tau value of 0.5856 (*p* < 0.000001; two-sided, from R cor.test), indicating a statistically significant relationship.

To investigate biologically significant changes in enhancer activity, we analyzed the expression of the enhancer RNA (eRNA) as a proxy for enhancer activation. We performed an integrative analysis combining ChIP-sequencing (ChIP-seq) to identify the location of 2040 T/N-SEDs) and RNA sequencing (RNA-seq) to define the RNA expression of such SEDs. Subsequently, we conducted a differential expression analysis of 2040 SED eRNA abundance estimates between 47 tumor and 25 normal samples (Figure 5). A volcano plot illustrating the results of differential eRNA expression analysis exhibited significant changes in expression in both tumors and normals. For illustration purposes, we annotated each SED eRNA with a gene closest to its center. Significantly differentially expressed SED eRNAs (log2 fold change >1.5 and adjusted *p* value <0.05) are shown in red on the volcano plot with notable examples of eRNA that regulated *CLDN1*, *TP63*, and *EGFR*, which displayed substantial upregulation in tumors compared to normal tissues. Conversely, eRNA that regulated genes such as *PAX5*, *BLK*, *MEF2C*, and *ECM1* exhibited downregulation in tumors. The significant eRNAs highlighted in the plot suggest potential regulatory roles of SED and their eRNA in tumorigenesis (Figure 5A). To study the relationship between eRNA and mRNA expression, we correlated log fold change of eRNA and mRNA for every gene with both values (*n* = 2040, Figure 5B). The Kendall’s Tau correlation coefficient equaled 0.5856 (*p* < 0.000001), indicating a positive correlation, suggesting a coordinated regulation between eRNAs and mRNAs, with differences observed between tumor and normal samples.

In conclusion, our analysis of eRNAs revealed significant expression changes between tumor and normal tissues, suggesting their potential role in HPV+ HNSCC. These findings highlight the importance of eRNAs in cancer biology and their potential as therapeutic targets.

### Super-enhancer-mediated regulation of key pathways

**Figure 6:**
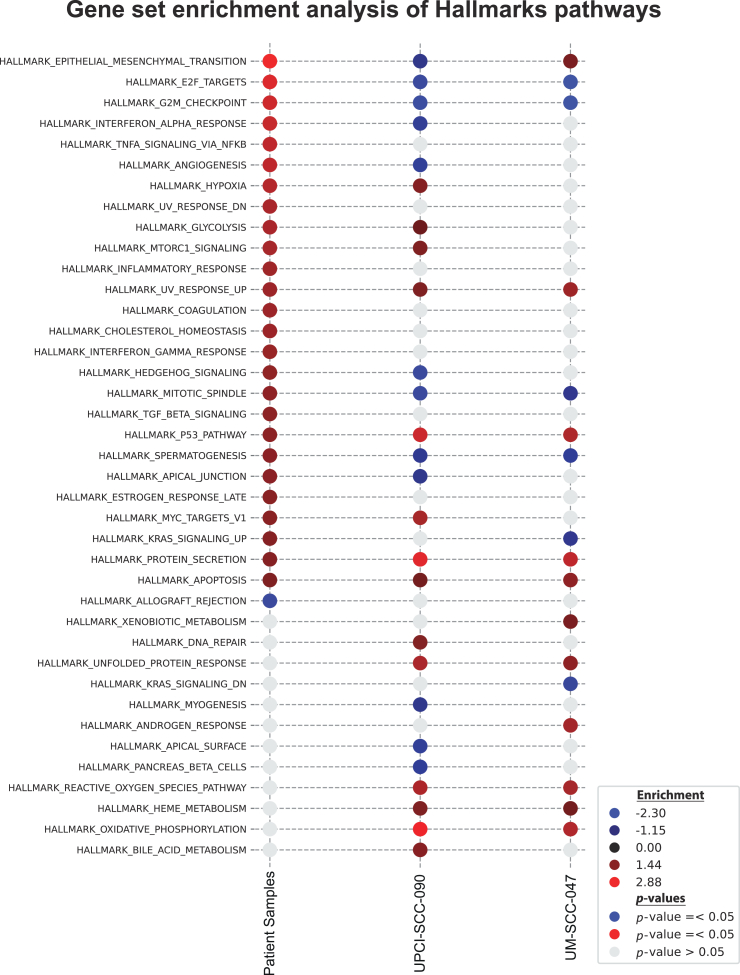
Gene set enrichment analysis of primary tissues and JQ1-treated cells The dot plot illustrates the results of gene set enrichment analysis using MSigDB Hallmark gene sets, comparing differentially expressed genes across patient samples (tumors vs. normal) and cell lines UPCI-SCC-090 and UM-SCC-047 after treatment with JQ1 (JQ1 vs. DMSO). Each row represents a specific Hallmark pathway; dot color encodes the normalized enrichment score (NES; red = positive enrichment, blue = negative), and gray dots indicate no significant enrichment (FDR-adjusted *p* value [p.adj, Benjamini-Hochberg] > 0.05), while colored dots indicate significant enrichment (p.adj ≤0.05). Color intensity reflects |NES| (stronger color = larger magnitude of enrichment).

To investigate the impact on the biological processes regulated by super-enhancers, we performed gene set enrichment analysis with MSigDB Hallmark gene sets.^66^ We used differentially expressed genes from patient samples and two JQ1-treated HPV+ HNSCC cell lines, UPCI-SCC-090 and UM-SCC-047 (Figure 6). This analysis revealed several key pathways significantly upregulated in HPV+ HNSCC patient samples and influenced by epigenetic structure: “Epithelial-Mesenchymal Transition,” “E2F Targets,” “G2M Checkpoint,” “Mitotic Spindle,” “Spermatogenesis”. Notably, these pathways were downregulated following BET inhibition with JQ1 in both cell lines.

Notably, “Interferon Alpha Response,” “Angiogenesis,” “Hedgehog,” “Apical Junction,” and “KRAS Signaling” were upregulated in tumor samples and downregulated in the UPCI-SCC-090 cell line following JQ1 treatment. Additionally, the “P53 Pathway,” “UV Response Up,” “Protein Secretion,” and “Apoptosis” showed significant elevation in primary tumor samples and both HPV+ HNSCC cell lines after JQ1 treatment. Furthermore, “Hypoxia,” “Glycolysis,” “MTORC1 Signaling,” and “MYC Targets V1” were upregulated in primary tumor tissues and remained elevated in the UPCI-SCC-090 cell line after JQ1 treatment.

This data indicate that cancer-specific chromatin structures significantly impact cellular processes, such as motility, invasiveness, DNA damage, and division by regulating key cellular pathways that promote division, differentiation, and proliferation. Additionally, the upregulation of pathways like hypoxia and glycolysis after JQ1 treatment suggests ongoing metabolic adaptation and potential drug resistance. Overall, the persistent upregulation of these pathways after JQ1 treatment highlights their importance in cancer cell survival and suggests potential targets for therapeutic intervention.

## Discussion

In this study, we explored the epigenetic landscape and gene expression dynamics in head and neck squamous cell carcinoma (HNSCC), specifically focusing on HPV-positive (HPV+) HNSCC. The modest but deeply profiled ChIP-seq cohort reflects unavoidable tissue-access limitations; nonetheless, precedent studies have drawn robust conclusions from similarly small numbers of HPV+ models.^67^^,^^68^ Our multi-modal integration (PDX + patient RNA-seq + cell-line perturbation) compensates for the limited N and firmly anchors biological inference.

Our data support the model that HPV+ HNSCC exhibits a form of viral enhancer-addiction,^69^ analogous to the super-enhancer dependency described in MYC-driven hematological malignancies.^22^^,^^29^^,^^70^ BET inhibition, through JQ1, used in our study, collapses a BRD4-centered SE network that sustains both viral oncogenes (*E6*/*E7*) and host drivers (*MYC*, *TP63*, *EGFR*, *RAD51*, and *TFAP2A*).^68^^,^^71^^,^^72^^,^^73^ This dual vulnerability offers a mechanistic basis for the distinctive clinical phenotype of HPV+ versus carcinogen-driven (HPV−) disease.^69^^,^^71^^,^^74^

Through a comprehensive workflow combining H3K27ac-ChIP-seq and RNA-seq analyses, we identified critical TFs and tumor-specific super-enhancer domains (T-SEDs) that regulate gene expression in HPV+ HNSCC, such as TP63, SMAD3, FOSL2, JUND, JUNB, KLF5, and TFAP2A. Some of those we have previously defined to be dysregulated in HNSCC, including STATs, NF-κB, AP1, p53,^42^ as well as TFAP2A, which we have shown to be associated with EGFR resistance.^16^ Others, such as FOSL1 and KLF4, were recently shown to play a central role in SE activation during HNSCC carcinogenesis.^75^^,^^76^ Our results, therefore, narrow the list of TFs controlled by the HPV-specific H3K27ac landscape and sensitive to BRD4 blockade.

It is important to note that we utilized HMcan^77^ for peak calling in our ChIP-seq analysis to reliably identify regions enriched with specific histone marks while accounting for sample-specific biases and technical variability. HMcan, an adaptation of the MACS2 algorithm, has been optimized for analyzing ChIP-seq data from cancer genomes, often presenting significant copy number variations (CNVs). A key strength of this tool is its ability to account for CNVs and GC content, thereby enabling accurate distinction between true biological signals and genetic artifacts in cancer cells. We processed the filtered and sorted BAM alignments for each sample and used the corresponding input DNA as a control, allowing HMcan to minimize background noise and enhance peak detection sensitivity effectively. The application of HMCan in our study provided a robust framework for identifying regions with significant histone mark enrichment, leading to a more precise understanding of the regulatory landscape within HPV+ head and neck cancer.

The cistrome-based analysis revealed a distinct set of TFs associated with epigenetic domains in HPV+ HNSCC. Notably, TFs such as TP63, FOSL1, and JUND were enriched in tumor-specific enhancer and super-enhancer domains, indicating their crucial role in tumorigenesis. TP63 has been extensively studied and is known to play a pivotal role in squamous cell carcinoma by regulating genes involved in proliferation and survival.^78^ Indeed, computational analysis of the chromatin data have proposed a critical role of the TP63 transcription factor in the differentiation and development of human squamous cell carcinoma.^79^ That data were supported by other studies on mice cell lines, demonstrating its role in enhancer-binding and regulation.^80^ Additionally, TP63 was found to enhance the invasion properties of HPV+ keratinocytes.^81^ Moreover, analysis of the human HNSCC cell lines suggests that KLF4 and ETS1 TFs play an essential role in HNSCC enhancer regulation besides TP63.^76^^,^^82^ Moreover, the co-activation of the super-enhancer-driven CCAT1 by TP63 and SOX2 has been found to promote squamous cancer progression, revealing the interplay between these TFs in cancer.^83^

FOSL1 and JUND are also significant as they have been shown to drive metastasis and invasion in various cancers. FOSL1 is a well-known TF that regulates miR-21-5p expression by interacting with MIR21-associated super-enhancer (MIR21-SE), promoting the malignant progression of HNSCC.^84^ Zhang and colleagues also uncovered a novel SE-driven transcription mechanism involving FOSL1 as a key regulator within the AP-1 complex, which predominantly functions via selective associations with mediators to establish super-enhancers at a cohort of cancer stemness and pro-metastatic genes, such as SNAI2 and FOSL1 itself, thereby promoting tumor initiation and metastasis in HNSCC.^75^

TFAP2A significantly regulates cancer biology by influencing cell proliferation, differentiation, and survival.^85^^,^^86^^,^^87^ Our findings show that TFAP2A is enriched in promoter and enhancer domains in HPV+ HNSCC, suggesting a dual role in gene regulation. It binds enhancer regions modulating oncogenic genes like EZH2, which is involved in chromatin remodeling and facilitating transcriptional activation.^86^ TFAP2A modulates key oncogenes, including EGFR, and contributes to therapy resistance by promoting epithelial-mesenchymal transition (EMT) and cancer stem cell properties. We observed that TFAP2A upregulated shortly after initiating anti-EGFR therapy, implicating it in resistance mechanisms.^16^^,^^88^ Furthermore, its regulation of cell cycle and apoptosis genes underscores its therapeutic potential. In our study, TFAP2A is overexpressed in tumors versus normal tissues, and its expression decreases following BRD4 inhibition with JQ1, indicating sensitivity to epigenetic modulation. Thus, targeting TFAP2A, possibly with BRD4 inhibitors like JQ1, may be a promising therapeutic strategy in HPV+ HNSCC.

Another group using a genome-wide landscape of active enhancers in an HNSCC mouse model detected the involvement of potential TFs, predicted with motif analysis, and identified AP-1 as one of the critical oncogenic TFs in many cancers, including HNSCC.^89^ Transcriptomic and epigenomic data analysis showed that AP-1 and histone modifications coordinately regulate target gene expression in HNSCC.^89^ JUND, another AP-1 transcription factor, has been shown to contribute to the malignancy of several cancers by regulating cell proliferation and apoptosis. In particular, JUND has been identified as a crucial factor in Ras-driven lung cancer, suggesting a significant role in oncogenesis.^90^

We propose that HPV oncoproteins E6 and E7 cooperate with these master TFs (TP63, AP-1, and TFAP2A) to nucleate tumor-specific super-enhancer clusters in HPV+ HNSCC. This model aligns with reports of HPV– host “hybrid” enhancers that recruit BRD4 condensates^69^^,^^72^^,^^91^ to drive oncogenic transcription. Our data reinforce this concept by showing that BRD4 inhibition with JQ1 leads to direct repression of E6/E7 expression, implicating these viral oncoproteins as integral to the SE-regulatory axis. Future profiling using CUT&RUN or CUT&Tag, in combination with BRD4- or H3K27ac-anchored HiChIP or PLAC-seq, will be necessary to directly map cooperative enhancer nucleation and long-range looping in this context.

To validate our RNA-seq findings and assess the functional impact of BRD4 inhibition, we performed RT-qPCR for HPV16 *E6*/*E7* and key host genes in UM-SCC-047 and UPCI-SCC-090 cells following JQ1 treatment. *E6* and *E7* transcript levels were significantly reduced by 50%–70% in both lines (*p* < 0.01; Figure S2). *EGFR* expression was concurrently downregulated by 40%–60%, mirroring RNA-seq trends. *TFAP2A* was also significantly reduced by RT-qPCR in both cell lines, although RNA-seq showed strong repression only in UPCI-SCC-090. These findings validate that both viral and host oncogenic transcripts are sensitive to BRD4 blockade and support the functional role of SE-associated transcription in maintaining the oncogenic program.

The coordinated repression of *E6*/*E7*, *TP63*, *MYC*, *EGFR*, *RAD51*, and *TFAP2A* following JQ1 treatment highlights a therapeutically actionable SE-driven transcriptional network in HPV+ HNSCC. These findings provide compelling functional validation that the tumor-specific H3K27ac landscape is not only active but also druggable. They are consistent with prior studies demonstrating that BET inhibition displaces BRD4 from integrated HPV chromatin and collapses viral transcriptional hubs, mechanisms previously observed in cervical cancer models.^71^^,^^72^^,^^92^ Importantly, the absence of such enhancer architecture in normal mucosa suggests reduced BRD4 dependency in non-malignant tissue, supporting a potential therapeutic window; this, combined with the dual suppression of viral oncogenes and key host drivers, positions BRD4 inhibition as a uniquely attractive therapeutic strategy in HPV+ HNSCC. Indeed, JQ1 has been shown to be effective in reducing MYC-driven transcriptional programs in multiple myeloma, leading to tumor regression.^70^ This aligns with studies indicating that BET inhibitors like JQ1 can modulate gene expression in HNSCC, making them promising therapeutic agents.^93^

Moreover, JQ1 has shown efficacy in inhibiting tumor growth when combined with cisplatin in ovarian cancer^94^ and in combination with cetuximab in HNSCC, suggesting its potential for combination therapies in HNSCC.^16^ Given that EGFR and PI3K/AKT pathways remain actionable in HNSCC, these findings support the use of BET inhibitors in combination with anti-EGFR therapies or PI3K/AKT inhibitors, strategies that have shown preclinical synergy in HNSCC models.^95^^,^^96^ Furthermore, to better highlight this discovery, we note that the SE-91 core gene set, comprising genes upregulated in tumors, downregulated after JQ1, and located near tumor-specific SEs, offers a potential biomarker panel for identifying patients most likely to benefit from BET-directed therapies.

Our findings indicate that targeting the epigenetic landscape, particularly super-enhancers and associated TFs, could provide new therapeutic strategies for HPV+ HNSCC. The distance analysis of tumor-specific super-enhancer domains (T-SEDs) revealed that genes located within close proximity to these domains exhibit higher variability in differential expression. This suggests that T-SEDs play a significant role in regulating the expression of nearby genes, contributing to the distinct gene expression profiles observed in tumors. Additionally, we have demonstrated that SED activity extends over at least 1.3 Mb, consistent with literature that highlights the significant role of these regions in controlling gene expression over long distances from the transcription site. Recent studies have identified super-enhancers as key regulatory elements in HNSCC, controlling the expression of critical oncogenes such as EGFR and MYC.^61^ The significant enrichment of T-SEDs near upregulated genes in patient samples further supports the central role of super-enhancer networks in HPV+ tumorigenesis.

To further assess the transcriptional activity of these SEs, we performed an integrative analysis of H3K27ac ChIP-seq and RNA-seq data to quantify enhancer RNA (eRNA) expression. This revealed a correlation between eRNA and mRNA expression changes, indicating coordinated regulation of enhancer-promoter interactions. Significantly upregulated eRNAs were identified near key oncogenes, including *CLDN1*, *TP63*, and *EGFR*, confirming transcriptional activation at these tumor-associated SEs. Conversely, eRNAs linked to *PAX5*, *BLK*, *MEF2C*, and *ECM1* were downregulated in tumors, consistent with repression of normal lineage-specific programs. These findings, together with prior reports that eRNAs can regulate their target genes by facilitating enhancer-promoter looping, such as MYC in colon cancer,^97^ suggest that eRNAs serve not only as markers of enhancer activity but also as mediators of oncogenic transcription. Recent research has further demonstrated the role of eRNAs in enhancing oncogenic transcription and their potential as therapeutic targets in HNSCC.^98^ Together, these results underscore the biological and translational relevance of eRNAs and support the functional importance of SE in HPV+ HNSCC.

The pathway analysis revealed a significant impact of JQ1 treatment on key biological processes in HPV+ HNSCC cell lines, emphasizing the role of super-enhancer-mediated regulation in tumorigenesis. The downregulation of pathways such as “Epithelial-Mesenchymal Transition,” (EMT) “E2F Targets,” and “G2M Checkpoint” following JQ1 treatment indicates that inhibition of BRD4 disrupts critical processes involved in cell proliferation, cell cycle progression, and metastasis in HNSCC cells. The observed downregulation of the “Interferon Alpha Response” pathway in the UPCI-SCC-090 cell line suggests a reduction in immune evasion mechanisms post-JQ1 treatment. Conversely, the upregulation of the “Angiogenesis” pathway in the same cell line may reflect complex compensatory mechanisms affecting the tumor microenvironment, warranting further investigation.

Another important finding was the significant elevation of the “P53 Pathway,” “UV Response Up,” “Protein Secretion,” and “Apoptosis” pathways after JQ1 treatment, suggesting that BRD4 inhibition may reactivate tumor suppressor functions of p53, leading to increased apoptosis and potential tumor regression. This is particularly significant for HPV+ HNSCC, where p53 function is often compromised. The reactivation of p53-mediated pathways presents a promising therapeutic strategy, especially in tumors with PI3K/AKT pathway activation and p53 loss. However, the persistent upregulation of pathways like “Hypoxia,” “Glycolysis,” “MTORC1 Signaling,” and “MYC Targets V1” after JQ1 treatment indicates ongoing metabolic adaptations that may contribute to therapeutic resistance. This suggests that while JQ1 effectively targets certain oncogenic pathways, cancer cells may continue to rely on metabolic reprogramming for survival. Therefore, combining epigenetic therapies with metabolic inhibitors could enhance treatment efficacy.

In conclusion, this study underscores the importance of the epigenetic landscape, particularly super-enhancer domains (SED), in regulating gene expression in HPV+ HNSCC. Our comprehensive analysis reveals key TFs and tumor-SEDs that drive oncogenesis, offering potential targets for therapeutic intervention. Applying epigenetic inhibitors like JQ1 presents a promising strategy to modulate gene expression and improve outcomes for patients with HPV+ HNSCC.

### Limitations of the study

While our study provides comprehensive insights into the epigenetic regulation and transcriptional dynamics in HPV+ HNSCC, there are limitations to consider. While informative, the use of cell lines and patient-derived xenografts may not fully capture the complexity of primary tumors. Additionally, the reliance on ChIP-seq and RNA-seq data necessitates further validation through functional assays, advanced imaging techniques, and clinical studies to confirm the therapeutic potential of identified targets. Another limitation of the present work is the reliance on bulk ChIP-seq/RNA-seq, which precludes single-cell resolution; ongoing single-cell assays aim to address this constraint. We also note that integrating high-resolution 3-D maps is a priority for future work to confirm physical enhancer-promoter interactions suggested by these TF-binding patterns. Looking ahead, several exploratory paths could extend this work. (1) High-density CRISPRi tiling across top tumor-specific SEDs could pinpoint indispensable enhancer modules. (2) Next-generation BET strategies, such as cereblon-recruiting BRD4 degraders^99^ (e.g., ZXH-3-26), merit evaluation in newly generated HPV-positive PDX models. (3) Joint single-cell ATAC-seq/RNA-seq and, where feasible, spatial transcriptomics would provide finer resolution of intra-tumor SE heterogeneity and immune crosstalk. Pursuing these avenues may refine therapeutic targets, uncover resistance mechanisms, and guide biomarker-driven trial design.^26^^,^^100^ Future research in HPV+ HNSCC should focus on the crosstalk between dysregulated TFs, SEs, and other epigenetic regulators to better understand disease pathogenesis. Integrating multi-omics approaches, including genomics, transcriptomics, and epigenomics, could provide deeper insights into the molecular landscape of HNSCC and identify new therapeutic targets. Continued efforts to understand the complexities of TF dysregulation and SE activation in HPV+ HNSCC will pave the way for innovative therapeutic strategies and improved patient outcomes.

## Resource availability

### Lead contact

Requests for further information and resources should be directed to and will be fulfilled by the lead contact, Daria A. Gaykalova (dgaykalova@som.umaryland.edu).

### Materials availability

This study did not generate new unique reagents.

### Data and code availability

• H3K27ac ChIP-seq (PDX/UPPP) are deposited at GEO GSE112021; primary tumor vs. normal RNA-seq at GSE112027; JQ1-treated cell line RNA-seq at GSE281308. All accessions are listed in the key resources table and will be publicly available as of publication.
• The code for this analysis pipeline (R and Ruby) is available at https://doi.org/10.5281/zenodo.17049925.
• Any additional information required to reanalyze the data reported in this article is available from the lead contact upon request.

## Acknowledgments

D.A.G. was supported by a 10.13039/100006381Research Scholarship grant, RSG-21-020-01-MPC, from the 10.13039/100000048American Cancer Society, United States; R01DE027809 and 1R01DE033426 from the National Institute of Health, and by a 10.13039/100017788ECOG-ACRIN 10.13039/100000043Cancer Research group award 30006561 funded by the 10.13039/100000054National Cancer Institute, United States. Assignment FFRW-2025-010 (125091010189-3) supported the Cistrome bioinformatics analysis. The TF-enrichment pipeline was supported by MSHERF under agreement no. 075-15-2025-484, within the Federal Scientific-Technical Program for the Development of Genetic Technologies (2019–2030).

## Author contributions

Conceptualization: F.T.Z., L.D., A.V.F., and D.A.G.; methodology: F.T.Z., S.S.C., M.D.K., E.J.F., I.V.K., L.D., A.V.F., and D.A.G.; investigation: F.T.Z. (conducted RNA-seq experiments on JQ1-treated cell lines), S.S.C., M.D.K., I.E.V., A.L., R.E., E.I., D.X.X., T.G., E.J.F., I.V.K., L.D., A.V.F., and D.A.G.; data curation and formal analysis: F.T.Z., S.S.C., M.D.K., I.E.V., A.L., R.E., E.I., D.X.X., T.G., E.J.F., I.V.K., L.D., A.V.F., and D.A.G.; project supervision: L.D., A.V.F., and D.A.G.; writing – original draft: F.T.Z., S.S.C., M.D.K., L.D., and D.A.G.; writing – review and editing: all authors.

## Declaration of interests

The authors declare no competing interests.

## STAR★Methods

### Key resources table

**Table:** 

REAGENT or RESOURCE	SOURCE	IDENTIFIER
**Antibodies**		

H3K27ac (Acetyl Lys27) Rabbit mAb	Cell Signaling Technology (CST)	Cat#8173; RRID:AB_10949503

**Biological samples**		

HPV+ HNSCC patient tumor samples	Johns Hopkins Tissue Core	IRB Protocol# NA_00036235

**Chemicals, peptides, and recombinant proteins**		

Small-molecule inhibitor – JQ1	Selleck Chemicals	Cat#S7110

**Critical commercial assays**		

SimpleChIP Plus Enzymatic Chromatin IP Kit	Cell Signaling Technology CST)	Cat#9005
mirVana™ miRNA Isolation Kit	Thermo Fisher Scientific	Cat#AM1560
High-Capacity cDNA Reverse Transcription Kit	Thermo Fisher Scientific	Cat#4368814
TaqMan Gene Expression Assays	Thermo Fisher Scientific	EGFR (Hs01076078_m1), TFAP2A (Hs01029413_m1), GAPDH (Hs02758991_g1)

**Deposited data**		

H3K27ac ChIP-Seq	N/A	GSE112021
RNA-Seq data	N/A	GSE112027
RNA-seq data of JQ1-treated cell lines	N/A	GSE281308
N/A	N/A	N/A

**Experimental models: Cell lines**		

UM-SCC-047 (HPV+ HNSCC)	Dr. Thomas Carey (University of Michigan)	RRID:CVCL_7759
UPCI-SCC-090 (HPV+ HNSCC)	Dr. Susanne Gollin (University of Pittsburgh)	RRID:CVCL_1899

**Software and algorithms**		

Analysis pipeline (R and Ruby)	This paper; Zenodo	https://doi.org/10.5281/zenodo.17049925
HMCan (ChIP-Seq peak calling)	Ashoor et al.^77^	RRID:SCR_010858
LILY (Super-enhancer identification)	N/A	https://github.com/BoevaLab/LILY
Salmon software (v. 0.14.1)	Patro et al.^101^	RRID:SCR_017036
DESeq2 package (v. 1.34.0)	Love et al.^64^	RRID:SCR_015687
GenomicRanges package (version 1.36)	Lawrence et al.^102^	RRID:SCR_000025
R (v. 4.1.0)	R Core Team^103^	RRID:SCR_001905
differential.coverage package (Version 0.2.0)	N/A	https://github.com/favorov/differential.coverage
fgsea package (v1.30.0)	Korotkevich et al.^104^	RRID:SCR_020938
msigdbr package (v7.5.1)	MSigDB^105^	RRID:SCR_022870
circlize	Gu et al.^106^	RRID:SCR_002141
ComplexHeatmap (v2.22.0)	Gu et al.^107^	RRID:SCR_017270
clusterProfiler (v4.14.6)	Yu et al.^108^	RRID:SCR_016884
org.Hs.e.g.,.db	Carlson, M^109^	RRID:SCR_024739
gencode19 (GENCODE)	Frankish et al.^110^	RRID:SCR_014966
Bowtie2 (version 2.3.5.1)	Langmead et al.^111^	RRID:SCR_016368
Samtools (version 1.9)	Danecek et al.^112^	RRID:SCR_002105
GTRD (Gene Transcription Regulation Database)	Yevshin et al.^47^	http://gtrd.biouml.org
GraphPad Prism (v10.2.3)	GraphPad Software	RRID:SCR_002798

### Experimental model and study participant details

#### Human participants

All tissue samples were collected from the Johns Hopkins Tissue Core under an approved IRB protocol (#NA_00036235). Primary tumor tissue samples were obtained from a cohort of 47 patients with HPV-associated oropharyngeal squamous cell carcinoma, as previously described.^113^ The cohort had a median age of 55 years (range 35–75) and was predominantly male (87%) and White (∼96%). Smoking history included 17 never, 12 current, and 17 former smokers. Tumor stages at diagnosis ranged from T1–T4 and N0–N3; ∼85% were stage III–IV. Normal controls comprised healthy oropharyngeal mucosa from uvulopalatopharyngoplasty (UPPP) specimens (*n* = 25; median age 27 years, range 18–51; 40% male; race 56% White, 36% Black, 8% other; mostly non-smokers, 76%), as reported previously.^113^

#### PDX models

Two patient-derived xenograft (PDX) models were generated from primary tumors from a 55-year-old male and a 65-year-old male (one with a 35 pack-year history); both were stage IV and have been described previously.^17^ Associated ChIP-seq data are available under accession GSE112021.

#### Cell lines

Human HPV+ HNSCC cell lines UM-SCC-047 (RRID:CVCL_7759) and UPCI-SCC-090 (RRID:CVCL_1899) were provided by Dr. Thomas Carey (University of Michigan) and Dr. Susanne Gollin (University of Pittsburgh), respectively. The cell lines were authenticated using the Short Tandem Repeat (STR) Profiling Service by the Johns Hopkins School of Medicine Genetic Resources Core Facility DNA Services.

### Method details

#### Cell culture and drug treatment

Human HPV+ HNSCC cell lines UM-SCC-047 (RRID:CVCL_7759) and UPCI-SCC-090 (RRID:CVCL_1899) were grown on high-glucose DMEM media (Clontech, Mountain View, CA, Cat# 11965092), supplemented by 10% fetal bovine serum (FBS; Atlanta Biologicals, Cat# S11550H) and 1% Penicillin-Streptomycin (Corning, Cat# 30-002-CI) at 37°C in 5% CO2. HPV+ HNSCC cell lines were cultivated in 6-well tissue culture dishes and then treated with 500 nM of JQ1, a bromodomain inhibitor (Selleck Chemicals, Houston, TX, USA, Cat# S7110) or 0.1% dimethyl sulfoxide (DMSO; Sigma Cat# D4540) as a vehicle control. The treated cells were incubated at 37°C for 72 h.^9^

#### H3K27ac chromatin immunoprecipitation (ChIP) and ChIP-Seq analysis

H3K27ac-specific ChIP-Seq data from 2 PDX samples (PDX1 and PDX2) and 2 UPPP samples (UPPP1 and UPPP2), previously published by our group were analyzed (GSE112021).^17^ Chromatin was extracted from these six samples, as previously described.^17^ The H3K27ac antibody (Cell Signaling Technology Cat# 8173, RRID:AB_10949503) was used to isolate DNA segments bound by this histone modification using SimpleChIP Plus Enzymatic Chromatin IP Kit (Cell Signaling Technology, Cat# 9005). Subsequently, we utilized Bowtie2 (version 2.3.5.1, RRID:SCR_016368) for mapping paired-end reads from the FASTQ files to the hg19 human reference genome (GRCh37/hg19), utilizing default parameters. Following alignment, the files were directly converted to sorted and indexed BAM format using samtools (version 1.9, RRID:SCR_002105). We utilized HMcan (version 1.31, RRID:SCR_010858) for peak calling, an adaptation of MACS2 (Model-based Analysis of ChIP-Seq algorithm). HMcan was applied to call ChIP-Seq peaks using the filtered and sorted BAM alignments for each sample, with the input DNA in that sample serving as a control.^114^^,^^115^

#### Super-enhancer, enhancer, and promoter identification in samples

To identify super-enhancers, typical enhancers, and active promoters, we applied the LILY^63^ software (https://github.com/BoevaLab/LILY) to the H3K27ac ChIP-Seq data, using the output from HMCan for each of the four samples separately. LILY identifies the strong peaks of the H3K27ac signal and classifies it into promoters (P) and enhancers (E). The software identifies the strongest subset of the enhancers and annotates them as super-enhancers (SEs). The LILY algorithm computes P/E/SE by analyzing the H3K27ac signal characteristics and their genomic location. Promoters (P) are defined as strong H3K27ac peaks located within a 2.5 kb window from the transcription start site (TSS) of annotated genes. Enhancers (E) are identified as peaks occurring outside this 2.5 kb window, typically in intergenic or intronic regions, indicating their role in regulating gene expression from a distance. Each enhancer region received a super-enhancer score corresponding to the sum of normalized H3K27ac density values (already corrected for copy number and GC content bias by HMCan). The regions were sorted according to the super-enhancer score, and the ROSE algorithm^30^ (RRID:SCR_017390) determined the threshold distinguishing typical enhancers from super-enhancers.

#### Domain identification

To ensure a non-ambiguous set, we utilized the promoters (P), enhancers (E), and super-enhancers (SE) identified by the LILY algorithm for regulatory domain identification. We defined regulatory domains by preparing a set of P/E/SE domains across two normal and two tumor PDX samples by concatenating all the overlapping P or E, or SEs (working with each category individually) from different samples. Thus, the intervals of the new annotation (from now on referred to as domains: promoter domains (PD), enhancer domains (ED), and super-enhancer domains (SED) contain all the positions covered by a P or E, or SE in at least one sample. In other words, we have pooled intervals for four ChIP-seq samples instead of sample-specific P/E/SE intervals to obtain a common geometry for all the samples. All further analyses were performed on the domain level.

#### RNA isolation, sequencing, and differential expression analysis

RNA-Seq data was obtained for the JHU cohort (GSE112027).^17^^,^^113^^,^^116^ In short, RNA-Seq libraries from ribosomal RNA depleted total RNA were prepared using the IlluminaTruSeq stranded total RNA Seq Gold kit and sequenced on the HiSeq 2500 platform sequencer (Illumina) and the TruSeq Cluster Kit. RNA sequencing data from both cohorts were normalized based on the version 2 protocols developed by TCGA.^7^ All downstream computational analysis was completed using R (v. 4.1.0, RRID:SCR_001905)^103^ unless otherwise specified. Gene expression values were quantified from RNA sequencing data using Salmon (version 0.14.1, RRID:SCR_017036). RNA from JQ1-treated and non-treated cell lines, as well as primary samples, were extracted using a mirVana kit (Thermo Fisher Scientific, Cat# AM1560). RNA-Seq data for this cohort was previously published.^9^ Results from Salmon were loaded into the R programming environment and analyzed by the DESeq2^64^ package (v. 1.34.0, RRID:SCR_015687). We performed the differential gene expression analysis comparing tumor samples to normal samples, as well as cell lines treated with JQ1 to those treated with the control (DMSO).

#### Quantitative PCR analysis of *E6*/*E7* and host oncogenes after JQ1 treatment

HPV-positive head and neck squamous cell carcinoma (HNSCC) cell lines UM-SCC-047 and UPCI-SCC-090 were treated with 500 nM JQ1 (Selleck Chemicals, Cat# S7110) or 0.1% DMSO as control for 72 h. Total RNA was extracted using the mirVana miRNA Isolation Kit (Ambion, Foster City, CA), and all RNA samples were reverse transcribed into cDNA using the High-Capacity cDNA Reverse Transcription Kit (Applied Biosystems, Foster City, CA). Quantitative real-time PCR (qRT-PCR) was performed using TaqMan chemistry on an Applied Biosystems 7900 Real-Time PCR System (RRID:SCR_018060), with reactions carried out in triplicate. Specific primers and probes were used to amplify the *E6* and *E7* regions of HPV type 16: for HPV-16 *E6*, the forward primer was 5′-TCAGGACCCACAGGAGCG-3′, the reverse primer was 5′-CCTCACGTCGCAGTAACTGTTG-3′, and the TaqMan probe was 5’-(FAM)-CCCAGAAAGTTACCACAGTTATGCACAGAGCT-3’; for HPV-16 *E7*, the forward primer was 5′-CCGGACAGAGCCCATTACAA-3′, the reverse primer was 5′-CGAATGTCTACGTGTGTGCTTTG-3′, and the TaqMan probe was 5’-(FAM)-CGCACAACCGAAGCGTAGAGTCACACT-3’. Host gene expression for *EGFR* (Hs01076078_m1) and TFAP2A (Hs01029413_m1) was quantified using TaqMan Gene Expression Assays (Applied Biosystems). All qRT-PCR experiments were normalized to *GAPDH* (Hs02758991_g1), and fold change in expression was calculated relative to DMSO-treated controls using the 2ˆ–ΔΔCt method.

#### Regulatory analysis of SEDs and mRNA

To determine the proximity of a gene to the nearest domain centers, we converted the input BED file containing domain coordinates into a GRanges object function from the GenomicRanges package (version 1.36, RRID:SCR_000025), and domain midpoints were calculated. Gene annotations were retrieved from the GENCODE gene model (gencode19, RRID:SCR_014966) using the differential.coverage package (version 0.2.0, released on Jun 9, 2021, https://github.com/favorov/differential.coverage). Using the distanceToNearest function from the same GenomicRanges package, we calculated distances from each gene transcription start site to the nearest domain center. We used the results from the DESeq2 differential gene expression analysis between tumor and normal samples (log2 fold changes and adjusted *p*-values) and the calculated distances to filter genes to include only those with adjusted *p*-values below 0.05 and distances less than 2 MB. The log2 fold changes of genes were split into two distributions according to the nearest SED, whether SED was tumor- or normal-specific, within a distance bin of 100 Kb (Figure 4). A ridge plot was generated to visualize the distributions of log2 fold changes across different distances, highlighting expression patterns between tumor and normal samples.

#### Transcription factor enrichment analysis

##### Map of genome-wide transcription factor binding sites

To test if the ChIP-seq domains were enriched for the binding site of particular transcription factors, we used the genome-wide map of binding sites, the cistrome,^48^ that was built from a large compendium of ChIP-Seq data, reprocessed in the Gene Transcription Regulation Database (GTRD, http://gtrd.biouml.org).^46^ In this study, we used only the reproducible cistrome regions, detected in alternative datasets or by different peak calling tools. Furthermore, we used the motif-supported cistrome subset, i.e., only the regions carrying significant binding motif occurrences (with motif P-value <0.0001). Neighboring regions bound by the same transcription factor were merged, and only regions from 50 to 10,000 bp were used in the analysis.

##### Estimating enrichment of TF binding sites in a set of domains

To identify whether the binding sites of particular transcription factors are enriched in disease-specific (T-tumor) and normal (N-normal) type of domains (T-SEDs, N-SEDs, T-EDs, N-EDs, T-PDs, N-PDs), we performed a comparison of all the domains of a specific type against control regions of the same lengths, that are located at a fixed distance from each domain. All 6 domain sets were assessed separately for each TF. For each domain, we created two control regions (so-called ‘shades’) of the same length at fixed distance D upstream and downstream. The control sets consisted of these control regions; overlapping regions within each control set were merged. The test regions (domains) and the control regions (shades) were separately intersected with the cistrome (see above) for each TF. To assess the statistical significance of the difference between overlaps of the domains and the control regions with the TF-specific cistrome, we utilized Fisher’s exact test on a 2x2 contingency table, counting the numbers of test and control regions with and without a nonempty overlap with the сistrome. The resulting P-values were corrected for multiple tested TFs using Bonferroni correction. Two values of D were tested (10,000bp and 100,000bp), and a lower odds ratio and less significant P-value from the Fisher’s test were selected each time from two results. We will refer to the odds ratio as the ‘TF enrichment’ for a TF in each type of domain. The TF was considered to be significantly enriched if the Bonferroni adjusted *p*-value was less than 0.05. To evaluate the difference between tumor- and normal-specific domains for significantly enriched TFs, we calculated the TF enrichment as the log odds ratio of the presence of TFBSs in the test regions (domains) compared to control regions (shades). We then calculated the difference between TF enrichment in tumor and normal samples as the delta between these log-transformed odds ratios. The positive delta means higher enrichment in tumor-specific domains, and the negative delta means higher enrichment in normal-specific domains.

##### TF expression

We generated custom gene lists to investigate TF regulation and assess the effects of JQ1 treatment. First, we calculated the TF enrichment in T- P/E/SED peaks (adjusted *p*-value less than 0.05 and delta more than 0). Briefly, we calculated odds ratios and *p*-values using the raw counts from the cistrome analysis on transcription factors (TFs) enriched in tumor super-enhancers domains (T-SEDs). We parsed the counts to calculate enrichment odds ratios for tumor and normal samples across P/E/SE using custom scripts (https://github.com/fernandozamuner/SE_HNSCC). Contingency tables were computed for 10k and 100k datasets, followed by a 2 × 2 × 2 comparison of odds ratios. The resulting *p*-values were merged and adjusted for multiple testing using the Bonferroni method. Enrichment plots of TFs in tumors against their enrichment in normal samples for P/E/SE were generated, highlighting significant TFs with adjusted *p*-values ≤0.05. Next, nodes were visualized to represent TFs associated with tumors specifically. Metadata was added to these nodes, including log2FC expression and adjusted *p*-values, derived from differential expression analyses comparing tumor and normal samples (47 tumor and 25 normal samples, as previously described) and differentiating between 2 HPV+ HNSCC cell lines, UM-SCC-047 and UPCI-SCC-090, treated with JQ1 or DMSO as a control. Using T-TFs, we created heatmaps of log2FC values for patients and cell lines post-JQ1 treatment.

##### Transcriptional characterization of the 91-gene core set via heatmaps and Gene Ontology enrichment

To characterize the transcriptional features of a core gene set (*n* = 91), we selected genes that met three criteria: (1) significantly upregulated in HPV+ HNSCC tumors versus normal tissue, (2) located near tumor-specific super-enhancer domains (T-SEDs) defined by H3K27ac ChIP-seq, and (3) downregulated following JQ1 treatment in both UM-SCC-047 and UPCI-SCC-090 cell lines. Log2 fold changes from these datasets were visualized using clustered heatmaps, constructed with ComplexHeatmap (v2.22.0, RRID:SCR_017270) and a symmetric green–white–red color scale defined via circlizecolorRamp2() (RRID:SCR_002141). To assess the statistical association between JQ1-downregulated genes and those near tumor-specific super-enhancers, Fisher’s exact tests were performed for each cell line (UM-SCC-047 and UPCI-SCC-090) as well as their intersection (core gene set). Contingency tables were constructed based on the presence or absence of genes in each category, relative to a defined background gene set representing all tested genes. Odds ratios, 95% confidence intervals, and *p*-values were computed using Fisher.test() function in R (v. 4.1.0, RRID:SCR_001905), and results were exported as.tsv and.xlsx files. To assess functional coherence within the core gene set, we performed Gene Ontology enrichment for Biological Process terms using enrichGO() (clusterProfiler v4.14.6, RRID:SCR_016884) with Entrez ID-mapped gene inputs supplied from org.Hs.e.g.,.db (RRID:SCR_004934). Dot plots of the top enriched terms were generated using dotplot() and saved as PDF.

##### eRNA analysis

We quantified eRNA expression by integrating ChIP-seq and RNA-seq data. In particular, we used the salmon software (v. 0.14.1, RRID:SCR_017036)^101^ to create an index over our SED (*n* = 2040) from the ChIP-seq data to create the map of SED that can express eRNA. Then, the estimated RNA expression of those SEDs (from now on, will be called eRNA) using the RNA-seq data. We performed differential expression (DE) analysis of such eRNA with the DESeq2 package (v. 1.34.0, RRID:SCR_015687).^64^ We converted the eRNA DE results to gene level by assigning the DE log fold change of an eRNA to the genes that overlapped with it within ±1.5 Mb. If a gene overlapped with multiple eRNAs, we calculated the mean log fold change. Also, for annotation purposes for each eRNA, we assigned a gene closest to the center of the SED/fragment corresponding to that eRNA and used that gene in the volcano plot.

##### Hallmark pathway analysis

We used the results of differential gene expression analysis of RNA-seq data from patients and cell lines for the gene set enrichment analysis. We obtained the Hallmark gene set from the Human Molecular Signatures Database (MSigDB) using the msigdbr package (v7.5.1, RRID:SCR_022870) and used log2 fold changes from the corresponding analysis as statistics for the fgsea package (v1.30.0, RRID:SCR_020938).

### Quantification and statistical analysis

All analyses were performed in R (v4.1.0, RRID:SCR_001905). Differential mRNA and eRNA expression were estimated with DESeq2 (v. 1.34.0, RRID:SCR_015687). DE models used ∼ condition (tumor vs. normal) and, for cell lines, ∼ treatment (JQ1 vs. DMSO). Wald tests were used to assess coefficients for each contrast in DESeq2, with Benjamini–Hochberg FDR control across genes per contrast. For genes, significance was defined as |log2FC| > 1 and FDR-adjusted *p*-value (Benjamini–Hochberg) < 0.05. For eRNAs, significance was defined as |log2FC| > 1.5 and FDR-adjusted *p*-value <0.05. Enrichment and overlap analyses (core 91-gene set and JQ1 response) used two-sided Fisher’s exact tests with odds ratios, 95% CIs, and BH-adjusted *p*-values reported; TFBS enrichment across transcription factors employed Bonferroni correction, with enrichment summarized as log odds ratios and tumor–normal differences reported as Δlog(OR). For distance-to-SED analyses, genes were binned in 100 kb increments (±2 Mb) and compared using two-sided Wilcoxon rank-sum tests; *p*-values across bins were adjusted using Benjamini–Hochberg. Gene Ontology enrichment used clusterProfilerenrichGO (BH adjustment; q < 0.05) and Hallmark pathway analyses used fgsea with BH-adjusted *p*-values (FDR <0.05). qRT-PCR data are mean ± SEM from ≥3 biological replicates; fold changes were tested versus a theoretical mean of 1.0 using two-sided one-sample t-tests (Wilcoxon signed-rank additionally where noted). Unless stated otherwise, all tests were two-sided. Significance symbols used in figure panels are: ∗*p* < 0.05, ∗∗*p* < 0.01, ∗∗∗*p* < 0.001 (adjusted where specified). For genome-scale analyses (RNA/eRNA DE, TF enrichment, distance-bin tests, GSEA), asterisks denote FDR-adjusted *p*-values (p.adj); for targeted assays (e.g., qRT-PCR), asterisks refer to nominal p unless noted. Volcano plots display log2FC versus −log10(FDR, “p.adj”). Figures/graphs were generated using R (v4.1.0, RRID:SCR_001905; packages listed in the key resources table) and GraphPad Prism (v10.2.3, RRID:SCR_002798).

## Supplemental information

Document S1. Figures S1 and S2

Table S1. List of super-enhancer regions detected by LILY, related to STAR Methods This table lists the super-enhancer regions identified using the LILY algorithm, specialized for detecting large clusters of enhancer elements with high transcriptional activity.

Table S2. Classification of super-enhancer domains (SEDs), related to Figure 2 This table categorizes super-enhancer domains into normal-specific (UPPP), tumor-specific, and non-specific (mixed) regions.

Table S3. Counts of transcription factor binding sites (TFBS) in tumor-specific and normal-specific domains, related to Figure 2 This table presents counts and statistical analyses of TFBS in promoter domains (PD), typical enhancer domains (ED), and super-enhancer domains (SED) for both tumor and normal samples. Metrics include odds ratios, the number of binding sites versus non-sites, negative log10 corrected significance values, and counts of sites within specific genomic distances (shades-10k and shades-100k) for each transcription factor.

Table S4. Enrichment analysis of transcription factor binding sites in tumor-specific domains, related to Figure 2 This table shows the results of the enrichment analysis of TFBS in tumor-specific PDs, EDs, and SEDs. It includes metrics such as delta values (delta10k, delta100k), *p* values (pval10k, pval100k), odds ratios for tumor and normal samples at different distances (tumorOR10k, normalOR10k, tumorOR100k, normalOR100k), adjusted *p* values (p.adj10k, p.adj100k), and overall analysis results for each transcription factor.

Table S5. Core gene set associated with tumor-specific super-enhancers and JQ1 sensitivity in HPV-positive HNSCC, related to Figure 3 Genes commonly upregulated in HPV+ tumors, downregulated by JQ1 in both UM-SCC-047 and UPCI-SCC-090 cell lines, and located adjacent to tumor-specific super-enhancer domains (T-SEDs) were identified through integrative analysis. The table includes gene symbols, Ensembl IDs, log2 fold changes in HPV+ tumors versus normal tissue (log2FC_patients), and log2 fold changes following JQ1 treatment in each cell line (log2FC_047, log2FC_090). These genes represent candidate transcriptional targets of BRD4-bound super-enhancers in HPV-associated HNSCC.

Table S6. Fisher’s exact test of association between JQ1-downregulated genes and tumor-specific super-enhancer domains, related to Figure 3 Fisher’s exact tests were performed to assess the overlap between JQ1-downregulated genes from UM-SCC-047, UPCI-SCC-090, and their intersection (core gene set) and genes located near tumor-specific super-enhancer domains (T-SEDs). The table reports odds ratios (OR), 95% confidence intervals (CI), and *p* values. All comparisons showed statistically significant association (p < 0.001), supporting a link between BRD4-inhibited transcriptional programs and super-enhancer proximity in HPV+ HNSCC models.

Table S7. Gene ontology biological process enrichment analysis of the 91-gene core set, related to Figure 3 The 91-gene core set, defined by upregulation in HPV+ tumors, downregulation by JQ1 in both UM-SCC-047 and UPCI-SCC-090 cell lines, and proximity to tumor-specific super-enhancer domains, was subjected to GO enrichment analysis. Over-representation analysis (ORA) was performed using the hypergeometric test (enrichGO, clusterProfiler), with fold enrichment, gene ratio, and adjusted *p* values calculated using the Benjamini-Hochberg procedure (padjustMethod = “BH”). The table lists the top significantly enriched Biological Process terms (adjusted p < 0.05).

Table S8. Differential gene expression near tumor-specific and normal-specific super-enhancer domains, related to Figure 4 This table provides Wilcoxon test p-values for differential gene expression between genes adjacent to tumor-specific and normal-specific SEDs across sequential 100 Kb distance bins up to 2 Mb.
