# Supplementary material for: Epigenetic profiling reveals key super-enhancer networks driving oncogenesis in HPV-positive HNSCC
Source: iScience. 2025 Oct 30;28(11):113911. doi: 10.1016/j.isci.2025.113911 (PMC12664489; doi:10.1016/j.isci.2025.113911)
Supplement: Document S1. Figures S1 and S2 [file mmc1.pdf]

iScience, Volume *28*

Supplemental information

Epigenetic profing reveals key super-enhancer networks driving oncogenesis in HPV-positive HNSCC

Fernando T. Zamuner, Spencer S. Chan, Michael D. Kessler, Ilya E. Vorontsov, Andrey Loginov, Rossin Erbe, Eddie Imada, Deborah X. Xie, Theresa Guo, Elana J. Fertig, Ivan V. Kulakovskiy, Ludmila Danilova, Alexander V. Favorov, and Daria A. Gaykalova

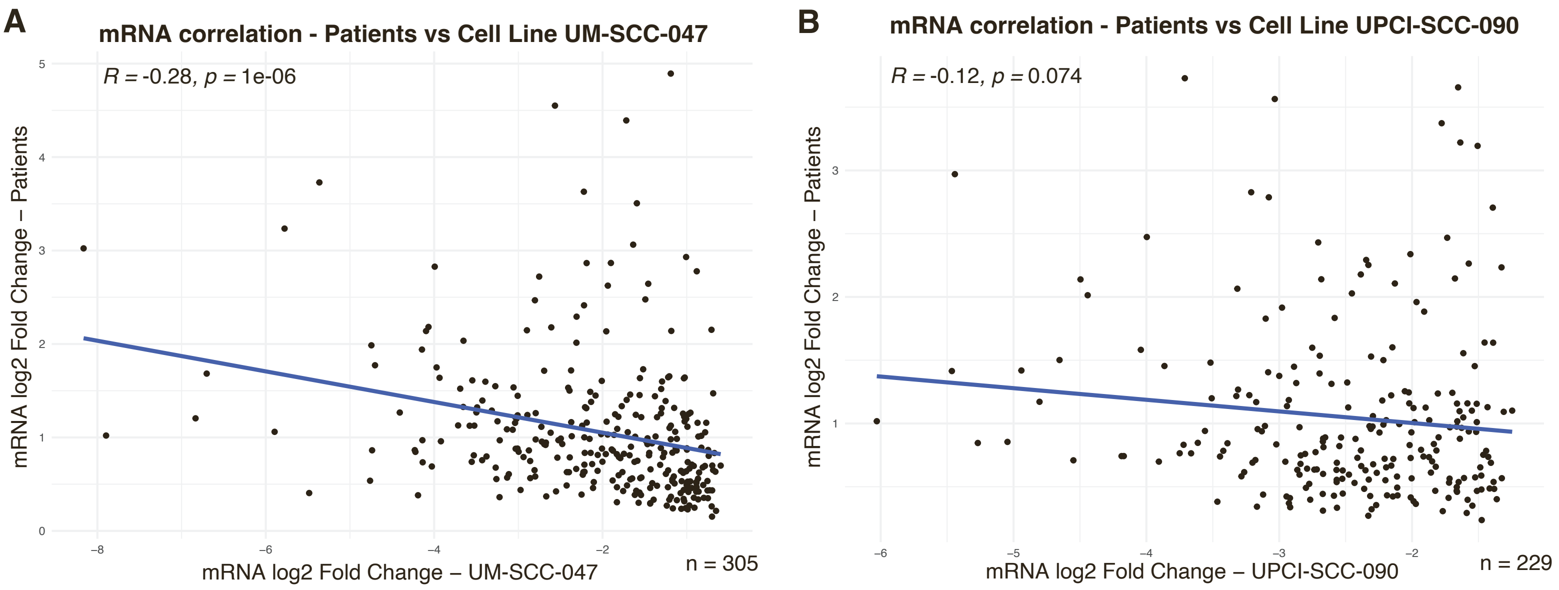

Supplementary Figure 1. Correlation of gene expression changes between HPV+ tumors and JQ1-treated cell lines, related to Figure 3. (A) Scatterplot showing a significant negative correlation (R = –0.28, p = 1e–6) between genes upregulated in HPV+ patient tumors and those downregulated following JQ1 treatment in UM SCC 047 cells (n = 305), consistent with suppression of tumor-associated transcripts by BRD4 inhibition. **(B)** In UPCI SCC 090 cells (n = 229), a non-significant negative correlation is observed (R = –0.12, p = 0.074), reflecting a modestly attenuated transcriptional response to JQ1 in this model.

JQ1 Inhibition Suppresses HPV (*E6* and *E7*) and Host Genes Involved in Proliferation and Signaling

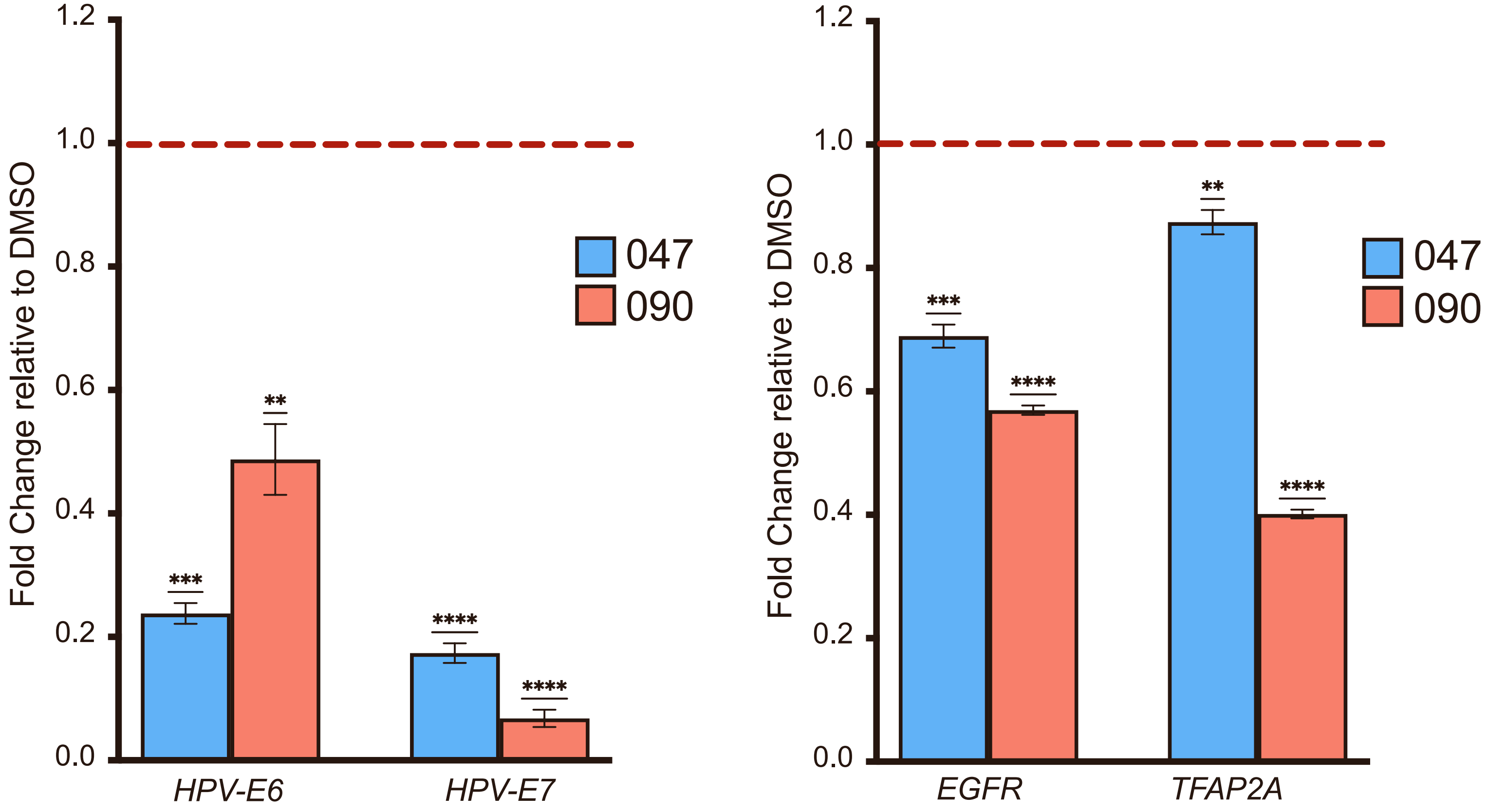

Supplementary Figure 2. JQ1 suppresses expression of HPV oncogenes and host transcriptional drivers in HPV-positive cell lines, related to STAR Methods. Relative expression levels of HPV oncogenes (*E6* and *E7*) and selected host genes (*EGFR* and *TFAP2A*) following JQ1 treatment in two HPV-positive HNSCC cell lines (UM-SCC-047 and UPCI-SCC-090). Expression is shown as fold change relative to DMSO-treated controls (dashed red line at 1.0). JQ1 significantly reduces *E6* and *E7* levels in both cell lines. Among host genes, *EGFR*, a key proliferative signaling driver, and *TFAP2A*, a transcription factor implicated in oncogenic regulation, are also significantly downregulated, with more pronounced effects observed in UPCI-SCC-090 cells. **Data are represented as mean ± SEM. Statistical significance was determined using a one-sample t-test and Wilcoxon signed-rank test relative to DMSO control (*$P < 0.05$; **$P < 0.01$; ***$P < 0.001$; ****$P < 0.0001$).
